# Historical trends in neuroanatomical tract-tracing techniques

**DOI:** 10.1007/s12565-025-00892-9

**Published:** 2025-08-24

**Authors:** Yasushi Kobayashi, Toshiyasu Matsui, Kiyomasa Nishii

**Affiliations:** 1 Department of Anatomy and Neurobiology, National Defense Medical College, Tokorozawa, Saitama 359-8513 Japan; 2 Laboratory of Veterinary Anatomy, Faculty of Veterinary Medicine, Okayama University of Science, Imabari, Ehime 794-8555 Japan

**Keywords:** Axonal transport, Degeneration, Neural pathways, Neurotropic viruses, Tract tracing

## Abstract

Numerous neuroanatomical tract-tracing techniques have been reported to demonstrate the origin, course, and termination of neural pathways. New techniques have been developed to achieve higher specificity and efficiency. Early tract-tracing studies at the microscopic level used non-specific staining, for example, by tracing fiber bundles of normal nervous tissue using myelin staining. However, when combined with neurodevelopment or degeneration, myelin staining provides important information regarding the major pathways, even in the early years. Impregnation techniques, including the Golgi method, have contributed to the demonstration of connections between individual neurons. Specific staining for degenerating myelin and axons has established most of the basic knowledge of the major pathways described in classical neuroanatomical textbooks. Since the 1970s, tract-tracing techniques using axonal transport have opened a new era of more detailed and selective connectivity analyses. They show normal morphology of neural pathways, including synaptic contact with target cells. Various tracer substances have been reported that can be injected into the nervous tissue and transported anterogradely or retrogradely through axons. Neurotropic viruses that can be transported trans-synaptically are particularly useful for analyzing the chains of neuronal connections. Introducing genes encoding tracer substances or reporters using various techniques, including electroporation, lipofection, and viral vectors, can yield higher intracellular concentrations of these molecules and provide high-contrast images of the entire dendritic tree and axonal arborization of labeled neurons. Since gene manipulation allows us not only to visualize neurons but also to control their functions, we can now conduct integrative research on neuronal morphology and function.

## Introduction

The nervous system is an organ system that specializes in information processing. It receives information from sensory organs and integrates it to produce output signals to muscles, glands, and other organs. Understanding the pathways for information processing in the nervous system is one of the central targets in basic and clinical neurology because the afferent and efferent connections of a neuron underlie its functions. Knowledge of the course of specific pathways provides a powerful tool for evaluating neurological symptoms caused by focal lesions in the nervous system.

**Fig. 1:**
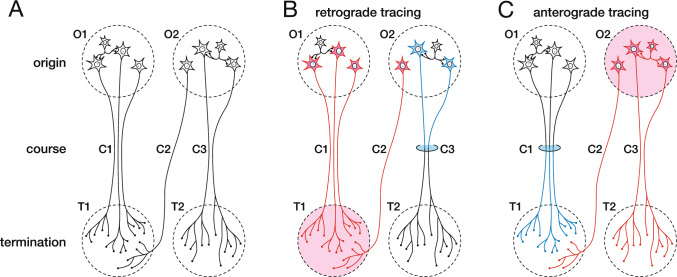
Basic concepts of tract tracing. **A** Components of neural pathways: origin (O1, 2), course (C1, 2, 3), and termination (T1, 2). **B** Retrograde tracing methods specifically visualize cell bodies with the same axonal target (pink) or passing location (blue). **C** Anterograde tracing methods specifically visualize axons and their terminals with the same nucleus of origin (pink) or passing location (blue)

However, neurons possess cell bodies ranging from a few microns in diameter to over 100 µm in the long axis, for Betz cells in the motor cortex of humans, and extend thin but sometimes very long axons exceeding 1 m in length. Discrepancies in cell diameter and axonal length often render it extremely difficult to trace pathways for information processing without specific visualization techniques. We used the term “tract tracing” to refer to the analysis of the origin, course, and termination of neural pathways (Fig. 1A).

In this review, we summarize the history of tract-tracing techniques to understand the trends in technical progress and their contributions to neurological science. Many techniques have been introduced over the long history of tract tracing (Table 1). In most cases, new techniques have been developed to achieve a higher specificity and efficiency. In early studies before the development of the degeneration methods, neural pathways were identified and traced using non-specific staining techniques. Although they were useful in describing major (i.e., massive and segregated) pathways, they were not able to discriminate specific pathways, particularly when axons of different origins and targets intermingled in their trajectory.

**Table 1:** Major technical progress in tract-tracing

nineteenth century	Discovery of Wallerian degeneration (Waller 1850) Macroscopic observation of retrograde degeneration (von Gudden 1870) Weigert method for myelin stain (Weigert 1882) Golgi method (Golgi 1885) Nissl method (Nissl 1894) Marchi method for degenerating myelin (Marchi and Algeri 1886)
1901–1950	Microscopic observation of retrograde degeneration (Nissl 1913) Glees method for degenerating axons (Glees 1946)
1951–1960	Nauta method for degenerating axons (Nauta and Gygax 1951)
1961–1970	Discovery of axonal transport of tritiated amino acids (Taylor and Weiss 1965)
1971–1980	HRP (Kristensson and Olsson 1971) Nuclear Yellow (Bentivoglio et al. 1980a, b) Fast Blue, True Blue (Kuypers et al. 1980)
1981–1990	WGA (Trojanowski et al. 1981) PHA-L (Gerfen and Sawchenko 1984; Ter Horst et al. 1984) FluoroGold (Schmued and Fallon 1986) Biocytin (Horikawa and Armstrong 1988) Carbocyanine dyes (Honig and Hume 1989) Neurotropic viruses (Kuypers and Ugolini 1990)
1991-	Isolectin B4 (Ambalavanar and Morris 1992) Viral vectors (Blömer et al. 1997; Chamberlin et al. 1998; Davidson et al. 2000; Kinoshita et al. 2002) Genetic tracing (Horowitz et al. 1999)

To address this problem, specific visualization techniques have been developed to positively stain a single pathway with a particular characteristic, such as cell bodies with the same axonal target or passing location (retrograde tracing; Fig. 1B) and axons and their terminals with the same nucleus of origin or passing location (anterograde tracing; Fig. 1C). Visualization efficiency is another important issue that must be addressed. If a pathway with a specific characteristic is visualized, techniques that can detect more neurons and their projections are preferred and are widely employed. In this review, we discuss non-specific staining, degeneration methods, and tract tracing using axonal transport and gene transfer techniques. We only describe the main techniques and elucidate the literature for each methodology. Readers interested in different techniques and their backgrounds are advised to refer to the following reviews (Lanciego and Wouterlood 2011, 2020; Nauta and Ebbesson 2012).

## Non-specific staining

### Myelin staining

**Fig. 2:**
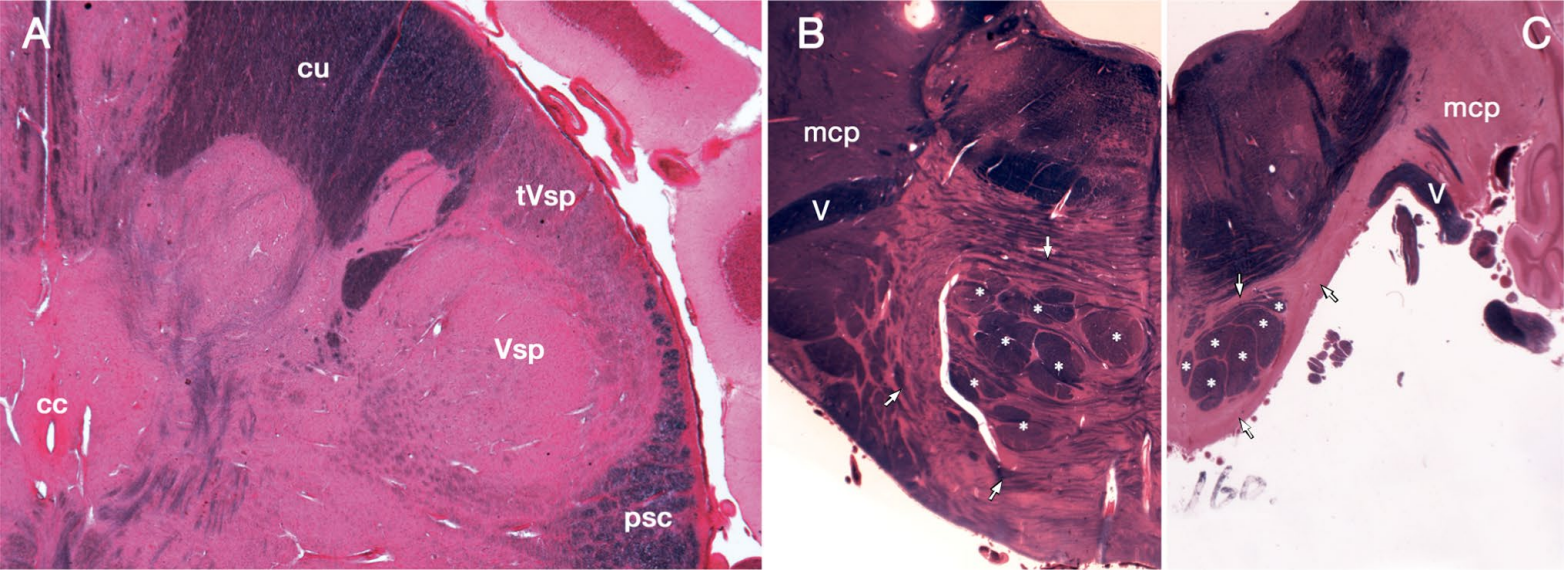
Tract-tracing using non-specific staining. **A** Dorsolateral part of the normal human lower medulla oblongata stained using the Weigert-Pal carmine method (Pal 1886). The difference in the thickness of myelin renders it possible to trace the course of the long fiber bundles. *cc* central canal, *cu* cuneate fasciculus, *psc* posterior spinocerebellar tract, *tVsp* spinal tract of the trigeminal nerve, *Vsp* nucleus of the spinal tract of the trigeminal nerve. **B** Pons of a normal adult human stained by the Weigert-Pal carmine method, which stains myelinated fibers in black and gray matter in red. **C** Pons of a patient with olivo-ponto-cerebellar atrophy. Degeneration of pontocerebellar projection neurons results in a marked reduction of the gray matter (area stained in red) in the ventral part of the pons. Due to the degeneration of pontocerebellar axons, myelinated fibers in the transverse pontine fibers (arrows) and middle cerebellar peduncle (mcp) are missing in panel C, whereas longitudinal pontine fibers look intact (asterisks). V: root of the trigeminal nerve

In the nineteenth century, myelin staining was utilized to detect neuronal pathways. Even in normal nervous tissue, myelin staining distinguished fiber bundles with different levels of myelination (different thicknesses or density of myelin; Fig. 2A). However, it is usually difficult to determine the exact origin or termination of the fibers. When neurons degenerate, their occupied area (nucleus or cortical layer) becomes atrophic, and their axons degenerate and lose myelin (Fig. 2B-C). If myelin staining is applied to such pathological cases, it can provide clues for tracing neural pathways. For example, Türck identified both ascending and descending fiber bundles in the spinal funiculi (Türck 1851). However, because small changes cannot be detected and precise terminations are not always clear, this method was soon replaced with specific staining for degenerating nerve fibers.

**Fig. 3:**
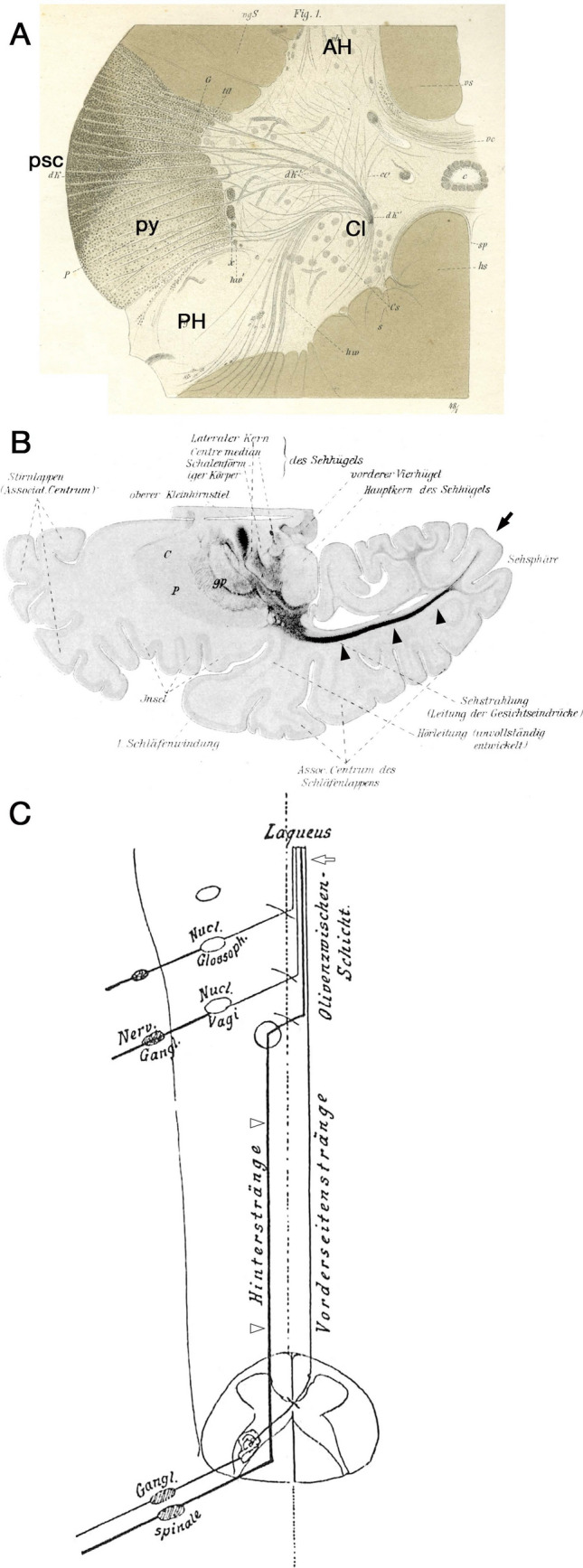
Tract tracing using myelogenesis. **A** Flechsig described the posterior spinocerebellar tract (psc) from Clarke’s column (Cl) based on myelogenesis. Note that the lateral pyramidal tract (py) is poorly myelinated. AH: anterior horn, PH: posterior horn. **B** Flechsig also demonstrated optic radiation (arrowheads) to the primary visual area (arrow) in the early stages of cerebral myelination. **C** Edinger described the spinal ascending tracts: posterior funiculus (arrowheads) and medial lemniscus (arrow)

Another condition that can be studied using myelin staining for tract-tracing is myelogenesis, that is, the development of myelin during developmental stages. Flechsig investigated myelogenesis using hematoxylin and gold-chloride staining and traced several tracts that myelinated earlier than their surrounding fiber bundles. He described the posterior spinocerebellar tract as the most dorsal portion of the spinal lateral funiculus (Flechsig 1876) (Fig. 3A). He traced the tract from Clarke’s column through the spinal cord, the medulla oblongata, and the inferior cerebellar peduncle to the cerebellum.

After the invention of the Weigert method for myelin staining (Weigert 1882) and its modification by Pal (Pal 1886) which consistently yielded higher-contrast images, Flechsig analyzed the myelogenesis of the cerebral white matter (Flechsig 1920). In Fig. 3B, the optical radiation is clearly visualized. Based on the time course of myelogenesis, he defined the association center (cortex) as the latest cortical area to be myelinated. Using myelogenesis and comparative neuroanatomy, Edinger demonstrated crossed ascending tracts from the spinal dorsal horn to the diencephalon, whereas the ascending fibers in the posterior funiculus were traced up to the ipsilateral posterior funicular nuclei in the medulla oblongata (Edinger 1889) (Fig. 3C).

Myelin staining has not been used in modern tract-tracing studies because of its non-specificity, which renders it difficult to differentiate adjacent fibers over long distances. Recent studies using polarized light imaging (Axer et al. 2011) and autofluorescent enhancement imaging (Costantini et al. 2021) may overcome these shortcomings and make it possible to trace individual myelinated fibers at very high resolution in a large volume of the nervous tissue.

#### Golgi staining

**Fig. 4:**
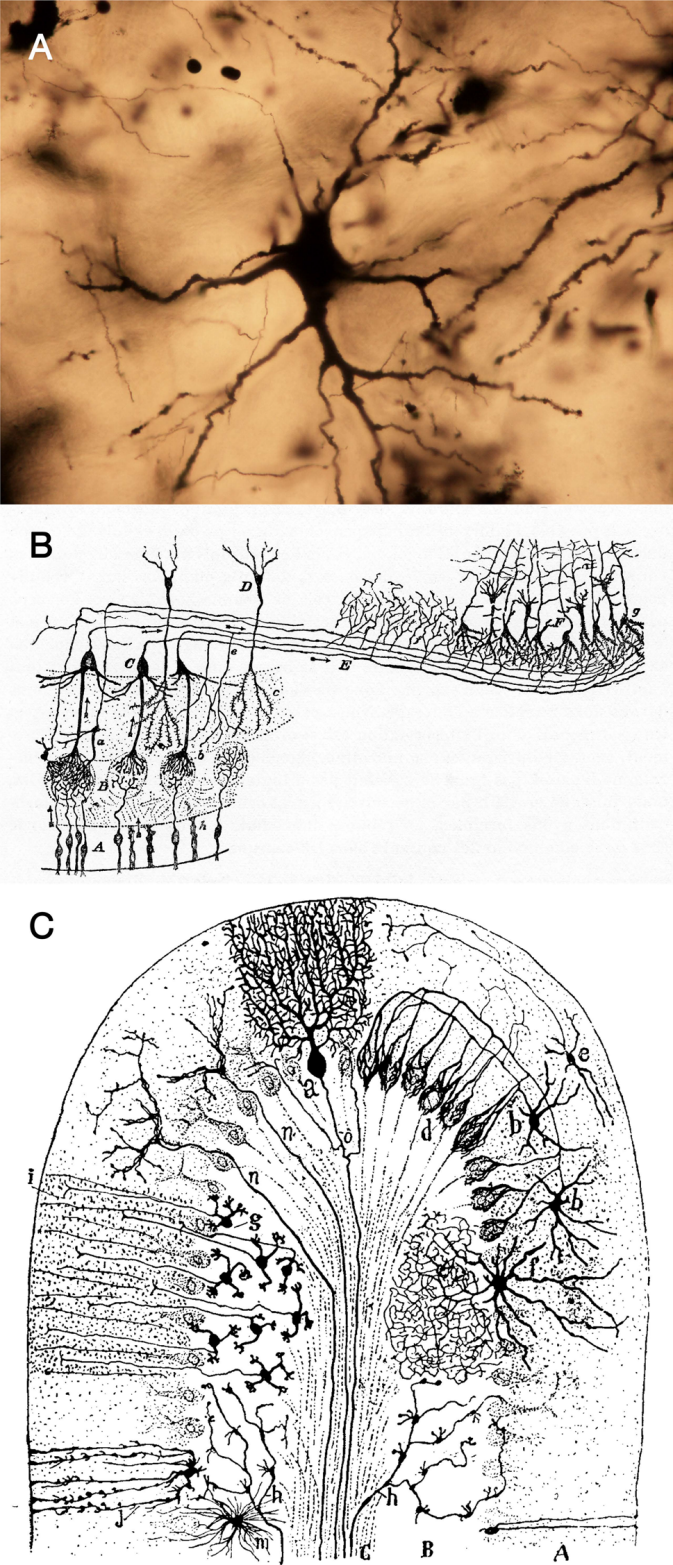
Silver impregnation. **A** Photomicrograph demonstrating a large neuron in layer VII of the spinal cord impregnated by the rapid Golgi method. **B** Intrinsic and extrinsic connections of the olfactory bulb illustrated by Ramón y Cajal. **C** Cerebellar cortical circuits are also established by Ramón y Cajal (1909)

Silver impregnation methods including the Golgi method and its modifications (Golgi 1885; Pannese 1999) are powerful tools for tract-tracing research. They do not visualize neurons with particular characteristics but instead randomly impregnate the cell bodies and processes of only a small population of neurons under optimum conditions (Fig. 4A). Sparse visualization often makes it possible to trace the entire extent of individual axons to terminal boutons. These methods are particularly effective for tracing short projections, such as intrinsic connections in the cortex. Ramón y Cajal established the principles of intracortical connections in the olfactory bulb and cerebellar cortex (Ramón y Cajal 1909) (Fig. 4B-C).

Although the non-specific staining mentioned above has been widely used and has contributed to the identification of neuroanatomical basics, it also has some limitations. Myelin staining cannot discriminate between axons of different origins or terminations when they intermingle at the same location (Fig. 1). Even with the Golgi method, which impregnates individual neurons, it is usually very difficult to trace long axons because identifying the same axon in adjacent sections is often problematic. The same axon must be identified in the next section, usually only by the relative position of the impregnated axons on the cut surfaces of the adjacent sections.

#### Degeneration methods

**Fig. 5:**
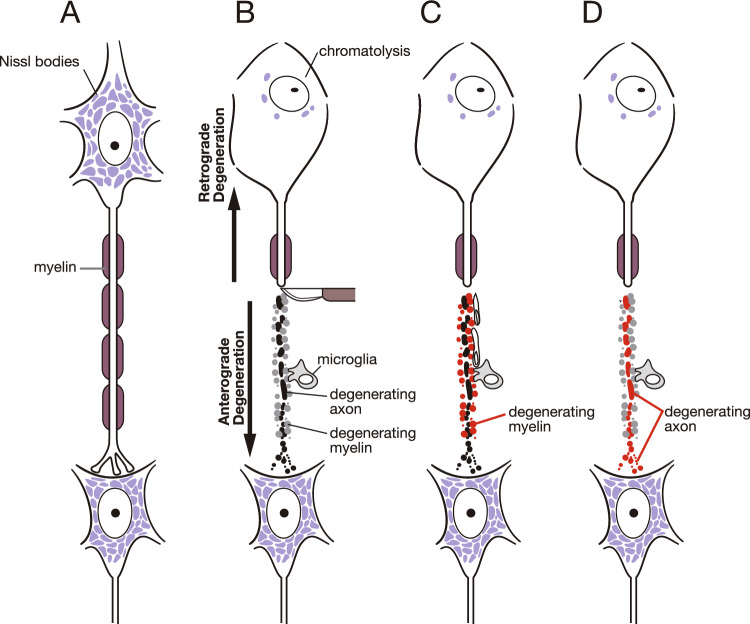
Schematic diagram showing tract tracing using degeneration methods. **A** A normal neuron sends an axon and makes synaptic contact with another neuron. **B** When an axon is damaged, the cell of origin swells and loses the staining of the Nissl substance (retrograde degeneration). The nucleus is displaced to the periphery of the cytoplasm. After this reaction, the neuron will degenerate and disappear or recover its original shape. The axon distal to the damage and its myelin degenerate (anterograde degeneration). **C** The Marchi method allows visualization of the breakdown products of myelin (red). **D** The Nauta and Glees methods allow visualization of a degenerating axon (red)

A more specific visualization of neural pathways was developed based on their degeneration. Once neurons are damaged, various reactions occur. Damage to cell bodies and axons can cause anterograde degeneration. Axons that are deprived of energy and molecular supply from their cell bodies undergo degeneration accompanied by the breakdown of their surrounding myelin (Wallerian degeneration) (Waller 1850). Axonal damage also causes retrograde degeneration, in which cell bodies swell and lose Nissl granules (chromatolysis) (Fig. 5A, B). Some cell bodies begin necrotic processes, whereas others survive and recover to normal conditions, depending on the severity of the damage.

#### Retrograde degeneration method

Macroscopically, von Gudden noticed severe atrophy of the thalamus after removal of the cerebral cortex (Von Gudden 1881), which prompted von Monakow to conduct microscopic confirmation of the atrophy of thalamic nerve cells (Von Monakow 1882). Franz Nissl, who invented cellular staining for nervous tissue using methylene blue (Nissl 1894) also described retrograde cellular changes in thalamic neurons after cortical ablation (Nissl 1913). Retrograde cellular degeneration was used to identify the cells of origin of a tract by creating lesions along its course or at its terminal regions in experimental animals. Immature animals are usually used for experiments because they show marked retrograde cellular changes (a modification of the Gudden method) (Brodal 1940b). It is particularly useful for demonstrating neurons projecting to the cerebral or cerebellar cortex because both cortices are located on the surface of the brain, and it is relatively easy to create discrete lesions without damaging the neighboring structures. Brodal et al. conducted a series of studies on neurons projecting to the cerebellar cortex, including those in the olivary nucleus (Brodal 1940a), pons (Brodal and Jansen 1946), and other brainstem neurons (Brodal 1954). Walker used retrograde degeneration to elucidate the thalamic neurons projecting to the cerebral cortex (Walker 1938).

However, retrograde degeneration methods do not always yield consistent results. In some cases, the cells of origin do not exhibit any discernible changes, even though their axons are disrupted. The survival of these neurons may be attributed to the existence of their intact collateral projections in other areas. Conversely, some cells exhibit degenerative changes without apparent axonal damage when their target neurons undergo retrograde degeneration.

#### Anterograde degeneration methods

**Fig. 6:**
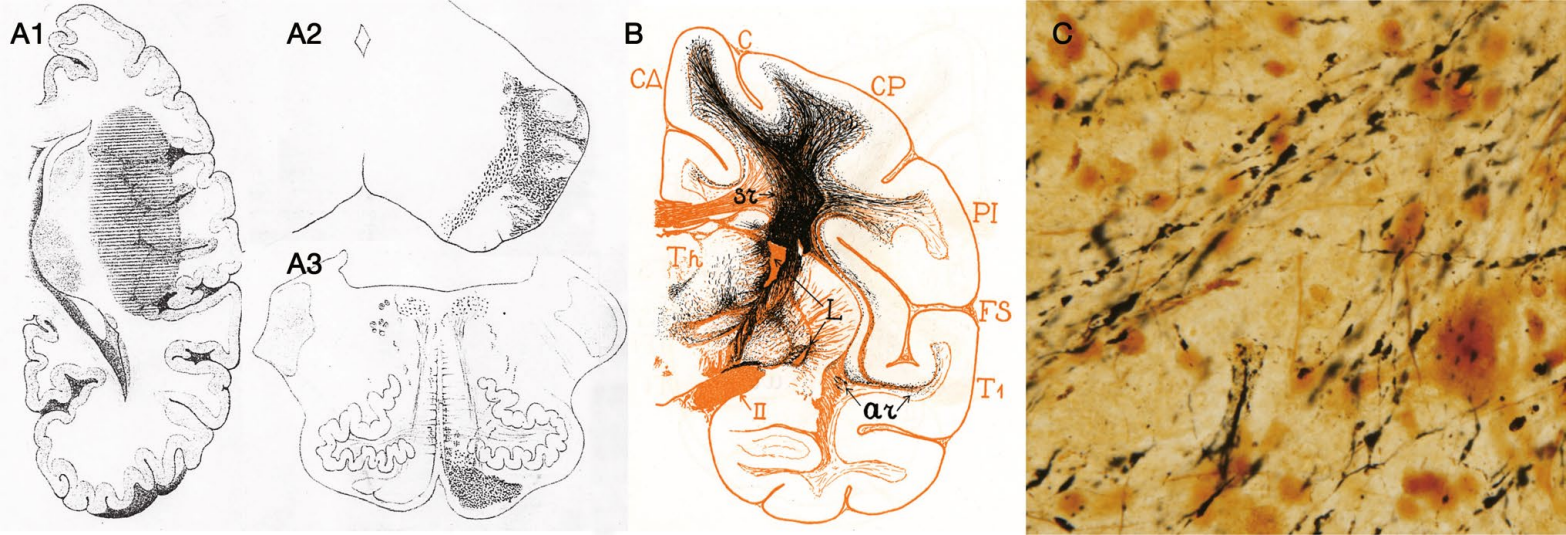
Tract tracing using degeneration methods. **A** Hoche traced the course of the pyramidal tract using the Marchi method (Hoche 1898). A1 is the horizontal section of the cerebrum showing the damaged area (horizontal stripe). A2 and A3 are the cross-section of the midbrain and medulla oblongata, respectively. Degenerated myelinated fibers are shown as black dots and lines. **B** Poliak illustrated degenerating fibers using the Marchi method in a monkey after lesions in the thalamus and globus pallidus (Poliak 1932). **C** Photomicrograph demonstrating degenerated axons and terminals in the cat thalamus after cortical lesions using the Nauta-Gygax method, which demonstrates black degeneration against a clear background

Degenerated fibers were effectively detected using specific staining for degenerated myelin and axons. Using osmium tetroxide, Marchi and Algeri developed a method for staining denatured myelin as black granules (Marchi and Algeri 1886) (Fig. 5C). Owing to the resulting high-contrast images, this technique has been widely employed in both experimental animals and human clinical cases to trace a wide variety of pathways. Hoche described the course of the human pyramidal tract and other descending fibers from the cerebral cortex after lesions in areas that received blood from the middle cerebral artery (Fig. 6A) (Hoche 1898). Walker provided comprehensive descriptions of thalamic afferents in monkeys using the Marchi method in the same monograph in which he determined the thalamocortical connections mentioned above (Walker 1938). Studies in monkeys (Poliak 1932) (Fig. 6B) and cats (Le Gros Clark and Boggon 1933a, 1933b) reported that various afferent connections distinguish specific areas of the cerebral cortex. Kuru used the Marchi method to demonstrate spinal ascending and descending tracts after cordotomy in patients with intractable pain caused by cancer (Kuru 1947, 1949). As he examined the sensory deficits after cordotomy, he also identified the sensory information carried by the damaged tract.

However, the Marchi method has some limitations. First, the optimum survival period varied among reports. The Marchi method detects fragments of degenerating myelin that strongly reduce the osmium tetroxide levels. Reactive substances seem to be different in the early and later stages after injury, and the procedure for optimal staining may differ (Strich 1968). However, these procedures cannot be used for extremely old lesions. Second, it cannot visualize non-myelinated fibers. Even myelinated fibers may be undetected where they lose myelin and give rise to terminal branches, limiting the Marchi method’s ability to accurately detect the areas of termination.

In the middle of the twentieth century, several methods were developed to visualize degenerating axons, including terminal ramifications (Glees 1946) (Fig. 5D, 6C). This enabled tracing of unmyelinated tracts and precise determination of the terminal area. Since the Glees method showed some false-positive staining in normal brains, the methods of Nauta and Gygax (Nauta and Gygax 1951, 1954) (Fig. 6C) soon replaced it. The Fink-Heimer method (Fink and Heimer 1967) also effectively impregnates degenerating axons and their terminal boutons. Although these methods certainly visualize degenerating axons, but not myelin, the visualized profiles in axons vary depending on the staining procedure; for example, the Nauta-Gygax method impregnates degenerating neurofilaments when omitting the pretreatment stages, but when the complete procedure is conducted, it impregnates the membranous structure of degenerating axons (Lund and Westrum 1966).

Degeneration methods have been used for both experimental and pathological damage. Thus, in humans, where experimental lesions or tracer injections are not permitted, degeneration methods can provide valuable information. However, experimental or pathological damage is not usually limited to a single nucleus, tract, or terminal area because axons of different origins intermingle with each other. Second, even fine damage may destroy adjacent regions directly or indirectly, for example, due to a disturbed blood supply in the areas downstream of the blood vessels in the lesion.

Various techniques for creating lesions have been developed to improve selectivity (Moore 1981). While physical lesions from simple cuts with a scalpel blade to thermal lesions and electrolysis may damage both nerve cells and fibers, the injection of neurotoxins such as kainic acid and ibotenic acid (Schwarcz et al. 1979) can selectively damage neuronal cell bodies but not passing fibers. This is particularly useful in the anterograde tracing of damaged fibers from the nucleus.

Another important limitation of the degeneration methods is that they cannot demonstrate the normal morphology of cell bodies or axons. Retrogradely degenerated cell bodies lose their normal sizes and morphologies. It is often difficult to identify constituent neurons in the affected nucleus or cortical layers. For axon terminals, we cannot demonstrate synaptic contact with target neurons, even if we can identify the terminal areas of the tract.

#### Axonal transport: anterograde, retrograde, and bidirectional.

**Fig. 7:**
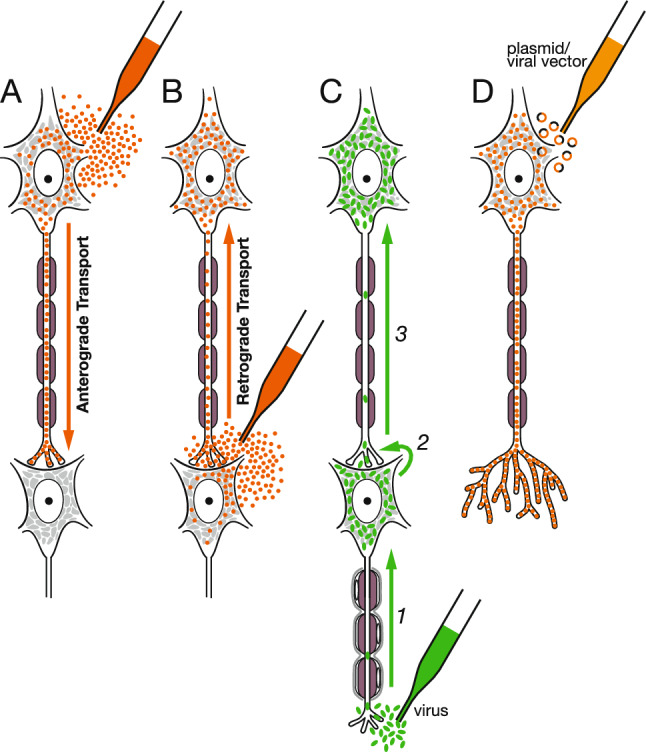
Schematic diagram showing tract tracing using axonal transport. **A** When anterograde tracers such as radioactive amino acids, BDA, and PHA-L are injected into the nervous tissue, neurons in and around the injection site take up the tracers and transport them through axons to terminals. **B** When retrograde tracers such as HRP, WGA, CTB, and fluorescent dyes are injected into the nervous tissue, axons or terminals take up the tracers and transport them through axons to the cells of origin. **C** Trans-synaptic labeling using neurotropic viruses. When rabies virus is injected, it is transported through axons to the cells of origin and is duplicated (*1*). Viral particles are taken up at synaptic terminals that contact the initially infected neurons (*2*) and are transported to the cell bodies (*3*). A chain of neuronal tracts can thus be demonstrated. **D** Tract tracing through the introduction of tracer or reporter genes using electroporation, viral vector, and other tools. Neurons produce these molecules for a long time and accumulate them in the cell body and processes, which visualizes the fine morphology of the entire neuron

To overcome these limitations, tract-tracing methods based on axonal transport have been developed. Neurons have a highly developed intracellular transport system that supplies necessary molecules anterogradely throughout neuronal processes and sends molecules retrogradely from the axon terminals to their cell bodies. Experimentally applied substances with neuronal affinity are internalized and transported intracellularly. A wide variety of tracer substances are injected into the nervous tissue or applied to the cut ends of peripheral nerves (Fig. 7A, B). Tracer injection methods include pressure injection, iontophoretic injection, and direct application of tracer crystals. After the optimal survival period for the tracer used, the animals are sacrificed, and the tissue is processed for visualization of the transported tracers. Processing methods depend on the chemical characteristics of the tracers, such as fluorescence, histochemical reactions, immunofluorescence, and immunohistochemistry.

**Fig. 8:**
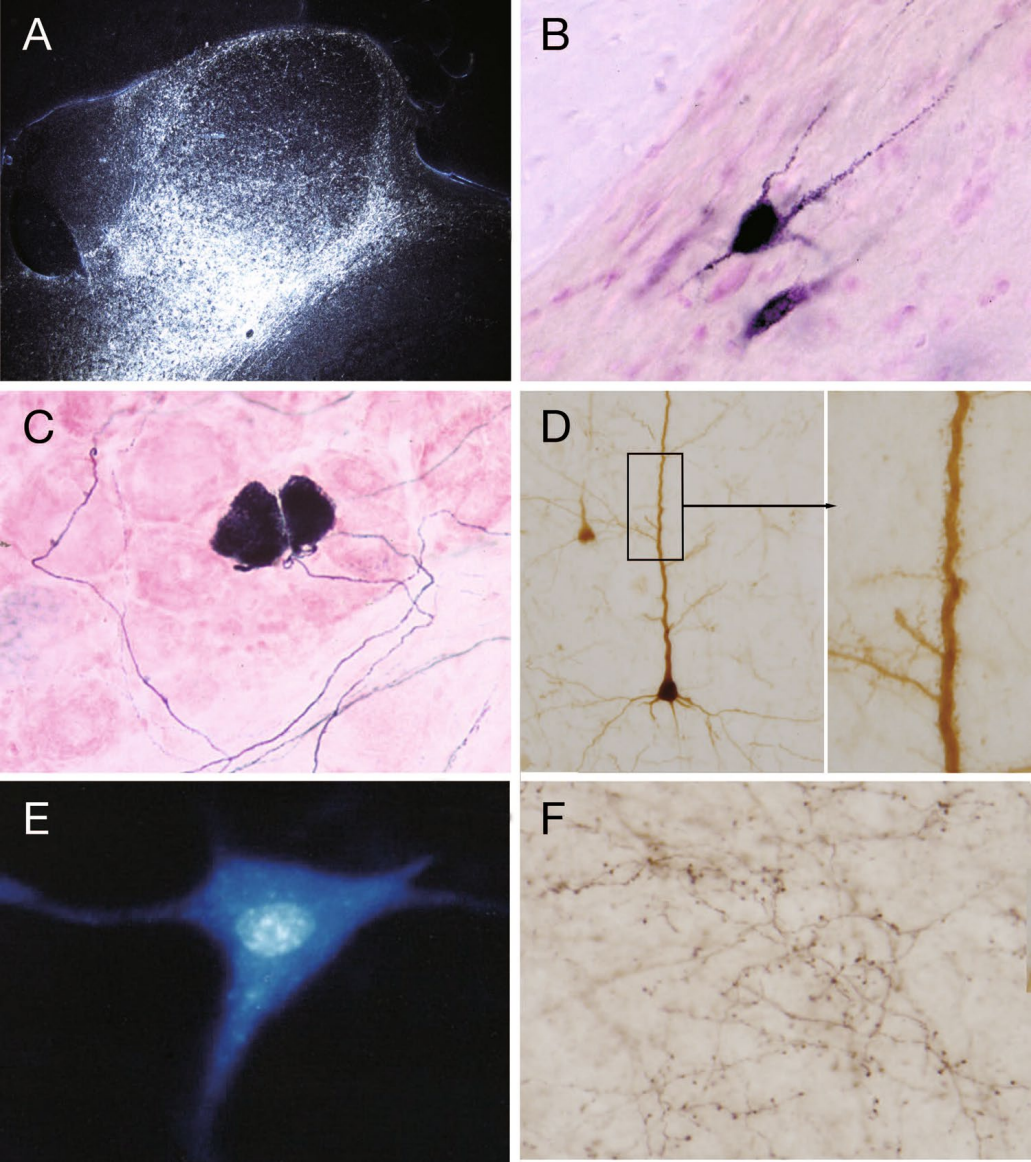
Labeling of tracers transported through axons. **A** Autoradiographic labeling viewed under dark-field illumination. An injection of tritiated amino acids in the macaque retrosplenial cortex resulted in marked anterograde labeling (brightly illuminated grains) in the anterior nuclei of the thalamus. **B** WGA-HRP labeling of a large neuron in the ventromedial part of the rat spinal dorsal horn after injection of WGA-HRP into the thalamic ventrobasal complex. **C** Selective labeling of a subpopulation of non-myelinated primary afferent neurons in the rat trigeminal ganglion using isolectin B4. **D** Retrograde labeling using CTB. Dendrites of labeled neurons are so clearly demonstrated that the dendritic spines can be identified. **E** Double labeling of fluorescent dyes: True Blue is deposited in the cytoplasm and Diamidino Yellow in the nucleus. **F** Anterograde tracing using BDA. Both axons and their terminal boutons as well as boutons en passant are visualized

The earliest substances used for tract tracing were radioisotope-labeled amino acids (typically, tritiated leucine and proline were preferred) (Cowan et al. 1972; Taylor and Weiss 1965). Once injected into the central nervous system, they are taken up by cell bodies, incorporated into newly synthesized proteins, and transported anterogradely through the axons. After an appropriate survival time (several days to 2 weeks), the tissue is processed using autoradiography to visualize the localization of radioactivity. Because background silver grains are inevitable in autoradiography, it is often difficult to differentiate weak labeling from the background. To obtain clear labeling, a sufficient amount of tritiated amino acids must be injected in the tissue. Consequently, this method is suitable for relatively large injections (more than 1or 2 mm in diameter) and for tracing the major projection pathways and terminal areas of neurons at the injection site (Fig. 8A). However, only silver granules in the emulsion resulting from photographic development could be observed and not the normal morphology of the labeled axons.

Horseradish peroxidase (HRP) was the first widely used molecule for retrograde tracing. It is taken up by neurons, transported through axons in both directions, and histochemically visualized using benzidine reactions (Kristensson and Olsson 1971). Owing to the relatively low rate of uptake and sensitivity of histochemical reactions, HRP is mainly used for retrograde tracing because its accumulation in the cytoplasm is easier to detect. In addition to its low efficiency, HRP is not suitable for long-term experiments because the survival period after injection is restricted to 3 days owing to the cytotoxicity of HRP. This may not be a problem in mice and rats but may result in false negatives for long pathways in larger animals.

Lectins are widely used to increase uptake efficiency. Lectins are carbohydrate-binding proteins that are highly specific for sugar groups of membrane-binding molecules. Each lectin binds preferentially to a specific carbohydrate. Wheat germ agglutinin (WGA) is the most frequently used lectin for tract-tracing (Trojanowski et al. 1981). WGA has been used for retrograde and anterograde tracing and is visualized using immunohistochemistry. WGA has also been used as a WGA-conjugated HRP and is detected histochemically (Fig. 8B).

The most frequently used lectin for anterograde tracing is *Phaseolus vulgaris* leucoagglutinin (PHA-L) derived from French beans (Gerfen and Sawchenko 1984; Ter Horst et al. 1984). It can be injected electrophoretically into a discrete area of nervous tissue and can clearly label the fine structures of axonal arborizations and terminals. Isolectin B4, derived from *Bandeiraea simplicifolia* (*Griffonia simplicifolia*), has specific affinity for subpopulations of unmyelinated primary afferent neurons (Ambalavanar and Morris 1992). When applied to the periphery, it is taken up by axon terminals and transported beyond the peripheral ganglion to the central nervous system. It can demonstrate both the cells of origin and the central projections of pain fibers from a small portion of the skin (Kobayashi and Matsumura 1996) (Fig. 8C).

Enterotoxins, such as cholera toxin subunit B (CTB) and heat-labile enterotoxin of *E. coli* (LT), are useful tools for improving neuronal affinity. While cholera toxin subunit A is responsible for toxicity, subunit B has an affinity for gangliosides on the cell surface but is not toxic. CTB is mainly used for retrograde tracing and can reveal the fine dendritic architecture of labeled neurons (Fig. 8D).

Fluorescent dyes, including Nuclear Yellow (Bentivoglio et al. 1980a), Fast Blue (Bentivoglio et al. 1980b), True Blue, Diamidino Yellow (Kuypers et al. 1980; Kuypers and Huisman 1984) and FluoroGold (Schmued & Fallon 1986), have also been used for tract tracing. These dyes are taken up by neurons and efficiently transported in both directions. Nuclear Yellow and Diamidino Yellow accumulate in the cellular nuclei, whereas True Blue and Fast Blue are deposited in the cytoplasm. Injections of Nuclear Yellow or Diamidino Yellow into one region and True Blue or Fast Blue into another enable the identification of double-projection neurons that send axonal collaterals to two different regions (Kuypers et al. 1980; Van Der Kooy et al. 1978) (Fig. 8E). Fluorescent dyes can be easily visualized using fluorescence microscopy without chemical or immunological reactions. FluoroGold can also be visualized immunohistochemically using specific antibodies.

Several other widely used tracer substances are available. Biocytin, a conjugate of D-biotin and L-lysine, has been used both for retrograde and anterograde tracing. It can be easily visualized using streptavidin-HRP or avidin–biotin-HRP complexes (Horikawa and Armstrong 1988). Related substances such as neurobiotin have been developed to reduce the toxicity and extend the half-life in living neurons (Lapper and Bolam 1991). Dextran amine and its derived compounds are also frequently used for retrograde and anterograde tracing, depending on their molecular weights (Schmued et al. 1990). Biotinylated dextran amine (BDA) is a biotin-containing compound that can be easily detected, similar to biocytin (Brandt and Apkarian 1992) (Fig. 8F). Dextran amines conjugated with different fluorescent dyes such as rhodamine (Glover et al. 1986; Schmued et al. 1990) and fluorescein (Novikova et al. 1997) are used to trace multiple tracts simultaneously in an animal, either using fluorescence microscopy or immunohistochemistry with specific antibodies for conjugated dyes.

Carbocyanine fluorescent dyes, including DiI (1,1ʹ-dioctadecyl-3,3,3ʹ,3ʹ-tetramethylindocarbocyanine perchlorate), DiO (3,3ʹ-dioctadecyloxacarbocyanine perchlorate), and DiA (4–4-dihexadecylaminostyryl-N-methyl-pyridinium), are often used for tract tracing during development (Blakemore & Molnár 1990; Godement et al. 1987; Honig & Hume 1986, 1989). Because they are lipophilic and dissolve not only the neuronal membrane but also myelin, the efficiency of neuronal labeling is much lower in adults, in which only a limited proportion of injected dyes are taken up by neurons. Carbocyanine fluorescent dyes can also be used for formalin-fixed tissues (Mufson et al. 1990). They dissolve in the membrane and diffuse along the axons. Because dyes are not actively transported through axons but move slowly by diffusion, they are particularly useful in the embryonic brain, where myelin is poorly developed and tracing for a short distance is sufficient to reveal the origin or target of the labeled axons (Godement et al. 1987). This is also useful in the human brain, where other tract-tracing methods using axonal transport cannot be applied. However, the subject of the tracing should be carefully selected because a recent survey of 61 published studies showed that the maximum tracing distance was 40 mm in the central nervous system and was mostly less than 10 mm (Mavrovounis et al. 2024).

#### Virus

The aforementioned tracers are deposited at the injection site, where their concentration is reduced by diffusion and uptake into neurons. If the tracers are reproduced in neurons, the labeling will be much more distinct, and the fine structures of the labeled axons and dendrites can be visualized. Neurotropic viruses are suitable for this purpose (Kuypers and Ugolini 1990). Rabies, pseudorabies, and herpes simplex viruses are often used as tracers (Kelly and Strick 2003). When a sufficient number of viruses are reproduced in infected neurons, they are often transmitted to neurons that make synaptic contacts with the initially infected neurons (Fig. 7C). This transneuronal labeling can demonstrate neural pathways composed of chains of neuronal tracts. Rabies virus is used for retrograde transneuronal tracing, whereas herpes simplex virus is used for anterograde transneuronal tracing (Dum and Strick 2013; Kelly and Strick 2003). Although these viruses are powerful tools for tract tracing, their toxicity to neurons renders it difficult to visualize the normal morphology of labeled neurons, particularly when a long survival period after the initial viral infection is necessary to reveal multiple chains of neuronal tracts. Genetic manipulation of viral tracers can make the virus less virulent or limit trans-synaptic spread, providing more control over the specificity and efficiency of tracing (Liu et al. 2022; Nassi et al. 2015).

#### Viral vectors

Viruses can also be used as vectors for gene transfer. If the genes of tracer molecules, such as WGA or fluorescent reporters, are transferred to neurons, the introduced molecules are produced for a long period and fill the interior of the neurons to visualize their fine structure (Fig. 7D). Genes are transferred using several methods, such as direct intracellular injection of plasmids, electroporation, or recombinant viruses. Viral vectors (typically adenovirus, adeno-associated virus, lentivirus, and Sindbis virus) are the most widely used vectors for tract tracing (Blömer et al. 1997; Chamberlin et al. 1998; Davidson et al. 2000; Kinoshita et al. 2002), and we can select among viruses with different characteristics, depending on the purpose of the experiments (Xu et al. 2020). Green fluorescent proteins and enhanced green fluorescent proteins are frequently used as reporters because they cannot only be detected by fluorescence immediately after sectioning the tissue but can also be visualized by immunohistochemistry to enhance the sensitivity and durability of the label. To obtain optimal images of the fine structures of axons and dendrites, modifications such as palmitoylation signals have been added to reporter proteins to affect intracellular protein localization to the membrane (Furuta et al. 2001). Even with traditional tracers, small but dense injections of tracer substances visualize detailed morphology of single neurons and their processes under the optimal conditions (Parent and Parent 2005). Viral vectors have extended the possibilities to trace the entire morphology of single neurons and their connections (see related studies by Kaneko and his collaborators) (Fujiyama et al. 2016, 2011; Furuta et al. 2011; Kuramoto et al. 2009; Matsuda et al. 2009). For details on the recent advances in relevant techniques, please refer to the review by Hioki et al. (2025) in this special issue.

If we also introduce genes that can control the activity of neurons, we can trace neural pathways and at the same time investigate the functional properties of the labeled neurons (Nectow and Nestler 2020). Isa et al. determined the projections of propriospinal neurons and their roles in manual dexterity in monkeys using retrograde tracing combined with a Tet-on system (Isa et al. 2022). Thus, morphological and functional analyses could be integrated.

#### Genetic tracing using transgenic mice

The development of genetic engineering has also enabled the labeling of neurons with specific gene expression (Horowitz et al. 1999). The introduction of reporter genes that produce tracer substances allows the visualization of the entire process of specific neurons without injecting tracers and avoids unnecessary damage to the tissue. However, neurons cannot be selectively labeled unless they possess specific promoters. To analyze the topography of the connections of a single type of neurons in one nucleus or cortical area, we still need localized injections of tracers or viral vectors to discriminate spatial differences in labeled neurons.

An approach called Brainbow was developed using Cre-lox recombination to stochastically express multiple fluorescent proteins in cells of the same tissue (Livet et al. 2007; Weissman et al. 2011). For the tract tracing, they label adjacent neurons with different colors to enable a more detailed analysis of local circuits than the Golgi method, in which all impregnated neurons show the same color. To date, different techniques have been developed to increase the number of colors and improve efficiency for analysis (Leiwe et al. 2024; Sakaguchi et al. 2018).

## Conclusion

We reviewed the long history of tract tracing to demonstrate how newer techniques have improved the specificity and efficiency of older visualization methods. Because we now have a wide variety of tracing techniques that can introduce genes that not only visualize neuronal pathways but also modify neuronal activities, the selection and combination of suitable tools are essential for conducting productive research on neural connections and their functions.
