# Correction: Historical trends in neuroanatomical tract-tracing techniques

**DOI:** 10.1007/s12565-025-00903-9

**Published:** 2025-10-29

**Authors:** Yasushi Kobayashi, Toshiyasu Matsui, Kiyomasa Nishii

**Affiliations:** 1 Department of Anatomy and Neurobiology, National Defense Medical College, Tokorozawa, Saitama 359-8513 Japan; 2 Laboratory of Veterinary Anatomy, Faculty of Veterinary Medicine, Okayama University of Science, Imabari, Ehime 794-8555 Japan

**Correction: Anatomical Science International (2025) 100:400-412**

10.1007/s12565-025-00892-9

In the sentence beginning ‘For details on the recent advances’ in this article, the text ‘review by Hioki et al. (2025)’ should have read ‘relevant review articles’.

